# Senescence-associated tumor growth is promoted by 12-Lipoxygenase

**DOI:** 10.18632/aging.203890

**Published:** 2022-02-14

**Authors:** Shilpa Patil, Jessica L. Reedy, Bradley T. Scroggins, Ayla O. White, Seokjoo Kwon, Uma Shankavaram, Alfonso López-Coral, Eun Joo Chung, Deborah E. Citrin

**Affiliations:** 1 Radiation Oncology Branch, Center for Cancer Research, National Cancer Institute, National Institutes of Health, Bethesda, MD 20892, USA

**Keywords:** senescence, radiation, senolytic, metastasis, Alox12

## Abstract

Radiation therapy is a commonly used treatment modality for cancer. Although effective in providing local tumor control, radiation causes oxidative stress, inflammation, immunomodulatory and mitogenic cytokine production, extracellular matrix production, and premature senescence in lung parenchyma. The senescence associated secretory phenotype (SASP) can promote inflammation and stimulate alterations in the surrounding tissue. Therefore, we hypothesized that radiation-induced senescent parenchymal cells in irradiated lung would enhance tumor growth. Using a murine syngeneic tumor model of melanoma and non-small cell lung cancer lung metastasis, we demonstrate that radiation causes a significant increase in markers of premature senescence in lung parenchyma within 4 to 8 weeks. Further, injection of B16F0 (melanoma) or Lewis Lung carcinoma (epidermoid lung cancer) cells at these time points after radiation results in an increase in the number and size of pulmonary tumor nodules relative to unirradiated mice. Treatment of irradiated mice with a senolytic agent (ABT-737) or agents that prevent senescence (rapamycin, INK-128) was sufficient to reduce radiation-induced lung parenchymal senescence and to mitigate radiation-enhanced tumor growth. These agents abrogated radiation-induced expression of 12-Lipoxygenase (12-LOX), a molecule implicated in several deleterious effects of senescence. Deficiency of 12-LOX prevented radiation-enhanced tumor growth. Together, these data demonstrate the pro-tumorigenic role of radiation-induced senescence, introduces the dual TORC inhibitor INK-128 as an effective agent for prevention of radiation-induced normal tissue senescence, and identifies senescence-associated 12-LOX activity as an important component of the pro-tumorigenic irradiated tissue microenvironment. These studies suggest that combining senotherapeutic agents with radiotherapy may decrease post-therapy tumor growth.

## INTRODUCTION

Radiation therapy is a commonly used curative treatment modality for cancer patients. Irradiation (IR) of tumors typically results in the simultaneous exposure of surrounding normal tissue to radiation, resulting in a host of changes such as oxidative stress, inflammation, activation of cytokine signaling, and extracellular matrix deposition [1–3]. These changes in normal tissue are implicated in late injury after radiotherapy. A substantial body of literature reports the effects of IR on tumor growth through alteration in immune infiltrate, cytokines, hypoxia, pH, and nutrient availability in tumor stroma [4–7].

Recently, cellular senescence in irradiated normal tissue has been implicated in late radiation injury [3, 8–10]. Cells that have undergone premature senescence due to stress, such as irradiation, are resistant to apoptotic cell death and effectively escape immune surveillance, resulting in their accumulation in tissue over time. Senescent cells contribute to further injury through depletion of normal tissue stem cells and via expression of the senescence associated secretory phenotype (SASP), a complex mixture of inflammatory, immunomodulatory, angiogenic, and mitogenic molecules secreted by senescent cells [11–18]. Expression of the SASP by senescent cells has been shown to propagate normal tissue injury and stimulate tumor growth [19]. Senescence in lung has been demonstrated to contribute to lung injury [3], and targeting senescence with agents that prevent senescence after injury or that selectively clear senescent cells (senolytic) have been shown to reduce lung fibrosis after radiation [3, 8, 10, 20, 21].

The mammalian target of rapamycin (mTOR) pathway is central in the regulation of cellular growth [22]. Pharmacologic inhibition of mTOR signaling elongates lifespan and delays cancer and age-related diseases [23, 24]. Although many mechanisms may contribute to the life span extending activities of mTOR inhibition, at a cellular level, inhibition of mTOR signaling has been demonstrated to prevent senescence in response to damaging stimuli [25, 26] and to inhibit the production of the pro-inflammatory senescence associated secretory phenotype (SASP) [19]. Inhibition of mTOR signaling has been shown to reduce radiation-induced premature cellular senescence *in vitro* and radiation-induced pulmonary parenchymal cell senescence *in vivo* [10].

Pulmonary radiation exposure results in a gene expression signature of aging and premature senescence in lung that corresponds to the accumulation of senescent cells [3]. One gene included in this signature, *Alox12* (*pl12-LOX* in humans and mice), encodes arachidonate 12-lipoxygenase (12-LOX), an enzyme that catalyzes peroxidation of arachidonic acid to 12S-hydroperoxyeicosatetraenoic acid (12S-HPETE), which is rapidly reduced to 12S-hydroxyeicosatetraenoic acid (12S-HETE) [27]. 12S-HETE has been previously identified as a mediator in inflammatory response, including upregulating NADPH oxidases [27, 28]. 12-HETE is secreted by senescent cells, and deficiency of 12-LOX has been shown to reduce radiation-induced premature senescence and lung injury [8].

To further explore the role of senescence in radiation-induced tumor growth, and to explore the role of senotherapeutics in mitigating this effect, we used a well-characterized pulmonary irradiation model that induces senescence in time dependent fashion. Using a lung experimental metastasis model, we analyzed the tumors that developed in the irradiated lungs at time points in which senescent cells were demonstrated to accumulate in irradiated lung. In parallel studies, prevention or clearance of radiation-induced senescence with senostatic agents targeting the mTOR pathway or a senolytic agent was capable of mitigating radiation-enhanced tumor growth. Radiation related expression of 12-LOX, a molecule previously implicated in radiation-induced senescence and lung injury [8], was reduced in irradiated lung tissue from mice treated with senostatic and senolytic agents. Experiments in mice deficient in 12-LOX indicated a crucial role played by this enzyme in regulating pro-tumorigenic microenvironment. Taken together, these studies demonstrate that IR-induced senescence in murine lung tissue is a significant contributor to tumor cell growth and colonization, and that this effect can be ameliorated with therapies targeting senescent cells.

## RESULTS

### Radiation enhances growth of experimental metastasis

**Figure 1:**
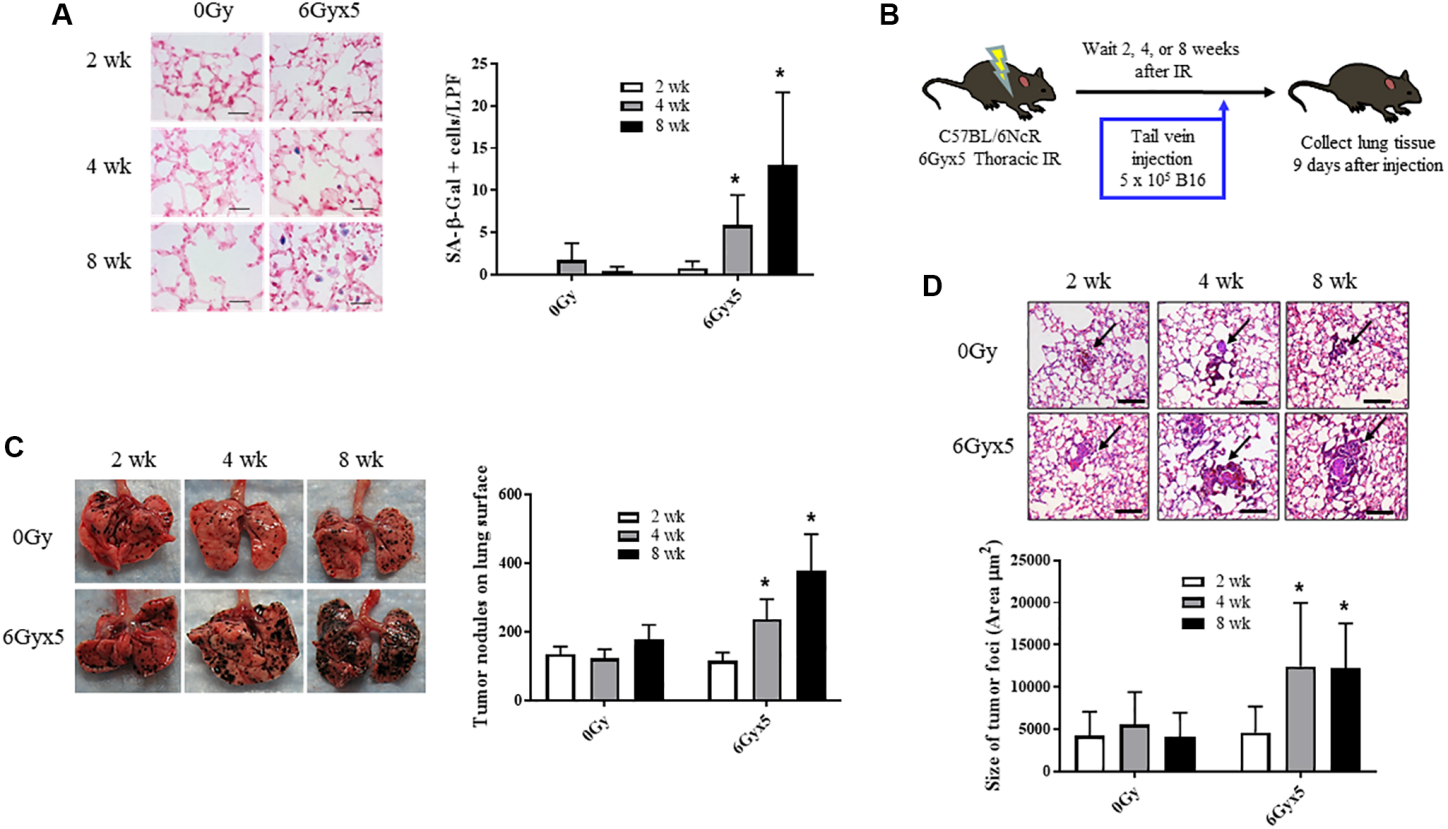
**Irradiation enhances growth of experimental tumor colonization.** Ten week old C57Bl/6NCr mice (*n* = 10) were irradiated with 5 daily fractions of 6 Gy (6Gyx5) to the thorax. (**A**) Lung tissue was collected at 2, 4, and 8 weeks after irradiation. SA-β-Galactosidase activity (blue) was evaluated in frozen lung tissue sections. (**B**) At 2, 4, 8 weeks after irradiation, 5 × 10^5^ B16F0 cells were injected intravenously via the lateral tail vein. Lung tissue was collected 9 days later to count tumor nodules on the lung surface (**C**) and to measure the size of tumor foci in the lung (**D**). Panel (**A**) scale bar 40 μm, panel (**D**) scale bar 100 μm. Figure **A**, **C**, **D**: ^*^ indicate statistical significance compared to 2 wk data from the same group. ^*^denotes *p* value <0.05.

To characterize the time dependence of radiation-induced normal tissue senescence in lung, mice were exposed to 5 daily fractions of 6 Gy (6Gyx5) thoracic IR and the number of cells exhibiting SA-β-Galactosidase activity was evaluated at 2, 4, and 8 weeks after IR (*n* = 10 per group). This dose regimen has been extensively validated as a method to induce fibrosis and senescence in irradiated lung [3, 8, 10, 21]. As previously described, in irradiated lungs, senescent cells increased significantly 4 and 8 weeks after IR compared to age matched unirradiated controls (Figure 1A). Based on these findings, a well characterized experimental metastasis model was used to evaluate the capacity of radiated lung to support tumor growth. B16F0 melanoma cells were intravenously injected 2, 4, and 8 weeks after IR and lung colonization was scored after 9 days (*n* = 10 per group, Figure 1B). Significantly more B16F0 tumor nodules were observed in irradiated lungs compared to unirradiated lungs at 4 and 8 weeks after IR (Figure 1C). Similarly, the area of tumor foci was significantly larger at 4 and 8 weeks after IR compared to foci in unirradiated age matched lung (Figure 1D). Confirmatory studies in the same model using a second murine tumor cell line, Lewis lung carcinoma, provided similar results (Supplementary Figure 1). These data demonstrate a correlation between the accumulation of senescent cells and tumor growth in irradiated lung.

To investigate possible alternative contributors to tumor colonization and growth, irradiated lung tissue collected at 8 weeks was further analyzed for several radiation related changes previously described at late time points (>16 weeks after radiation exposure) that could contribute to tumor growth, such as fibrosis, increased vascular permeability, and accumulation of alternatively activated macrophages [8, 29]. There was no significant difference in collagen accumulation in the lung at the 8 week time point (Supplementary Figure 2A). Although radiation increased the accumulation of F4/80+ cells (macrophages), there was no significant difference in the accumulation of Arginase-1+ macrophages at the 8-week time point (Supplementary Figure 2B, 2C). Vessel permeability is known to change in the early hours after thoracic irradiation [30] (confirmed in Supplementary Figure 2D), however later timepoints have not been assessed. Permeability assays conducted at the 8-week time point did not find significant differences in pulmonary vascular permeability between irradiated and unirradiated mice (Supplementary Figure 2E).

### Senostatic and senolytic therapy decreases senescence *in vitro*

Activation of mTOR signaling is characteristic of replicative, oncogene-induced, and stress-induced senescence [11]. Inhibition of either raptor-mTOR (TOR complex 1; TORC1) or rictor-mTOR (TOR complex 2; TORC2), as well as dual mTORC inhibition, prevents cellular senescence and restore proliferation potential [12]. Within this context, we sought to determine the capacity of a dual mTORC inhibitor INK-128 to prevent IR-induced cellular senescence. Rapamycin was included in the study considering its well-known senostatic abilities [31].

**Figure 2:**
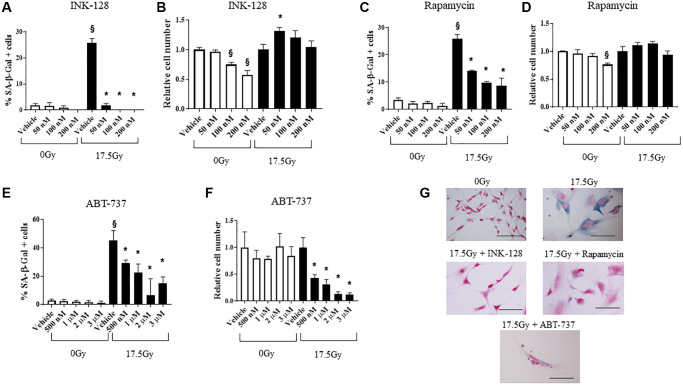
**Senostatic and senolytic action of drugs.** NIH-3T3 cells were treated with a single dose of radiation (0 Gy or 17.5 Gy). (**A**–**D**) Six hours after irradiation, cells were treated with INK-128 or Rapamycin. The percentage of cellular senescence, as determined by SA-β-Galactosidase activity, and the relative cell count were assessed five days after drug treatment. (**E**, **F**) NIH-3T3 cells treated with a single dose of radiation (0 Gy or 17.5 Gy). Three days after irradiation, cells were treated with ABT-737. Two days after drug was applied the percentage of cellular senescence, as determined by SA-β-Galactosidase activity, and the relative cell number were assessed. Relative cell counts for each drug is normalized to vehicle from the respective group (0 Gy or 17.5 Gy). (**G**) SA-β-Galactosidase activity of NIH-3T3 cells 5 days after irradiation and treated with vehicle, INK-128 (100nM), Rapamycin (100 nM) or ABT-737 (1μM) (Scale bar: 100 μm). ^§^: indicates *p* < 0.05 compared to 0 Gy, Vehicle. ^*^: indicates *p* < 0.05 compared to 17.5Gy, Vehicle.

NIH-3T3 and WI-38 fibroblasts (Figure 2, Supplementary Figure 3) were treated with 0 Gy or 17.5 Gy, followed by increasing concentrations of INK-128 or rapamycin 6 h after IR. The percentage of cellular senescence, as determined by SA-β-Galactosidase activity, and the relative cell number was assessed five days after drug treatment (Figure 2A–2D). Both INK-128 and rapamycin reduced the percentage of senescent cells after IR treatment while only causing minor reductions in relative cell numbers (Figure 2A–2D, 2G).

Senescent cells evade apoptosis by upregulating Bcl-xL and Bcl-w. Agents that inhibit these molecules are widely known to selectively induce apoptosis in senescent cells [32]. We confirmed the capacity of ABT-737, a BH-3 mimetic inhibitor of Bcl-2, Bcl-xL and Bcl-w [33] to selectively kill senescent murine cells *in vitro*. ABT-737 was delivered to NIH-3T3 cells 3 days after 17.5 Gy IR, and the percent of cells with SA-β-Galactosidase activity and relative cell numbers were assessed 2 days after drug treatment (Figure 2E–2G). ABT-737 significantly decreased the senescent cell population relative to vehicle alone at 5 days after IR with minimal toxicity in unirradiated cells. Together, these data demonstrate the senostatic function of rapamycin and INK-128, and senolytic activity of ABT-737 in murine cells.

### mTOR inhibition prevents IR-enhanced tumor growth

**Figure 3:**
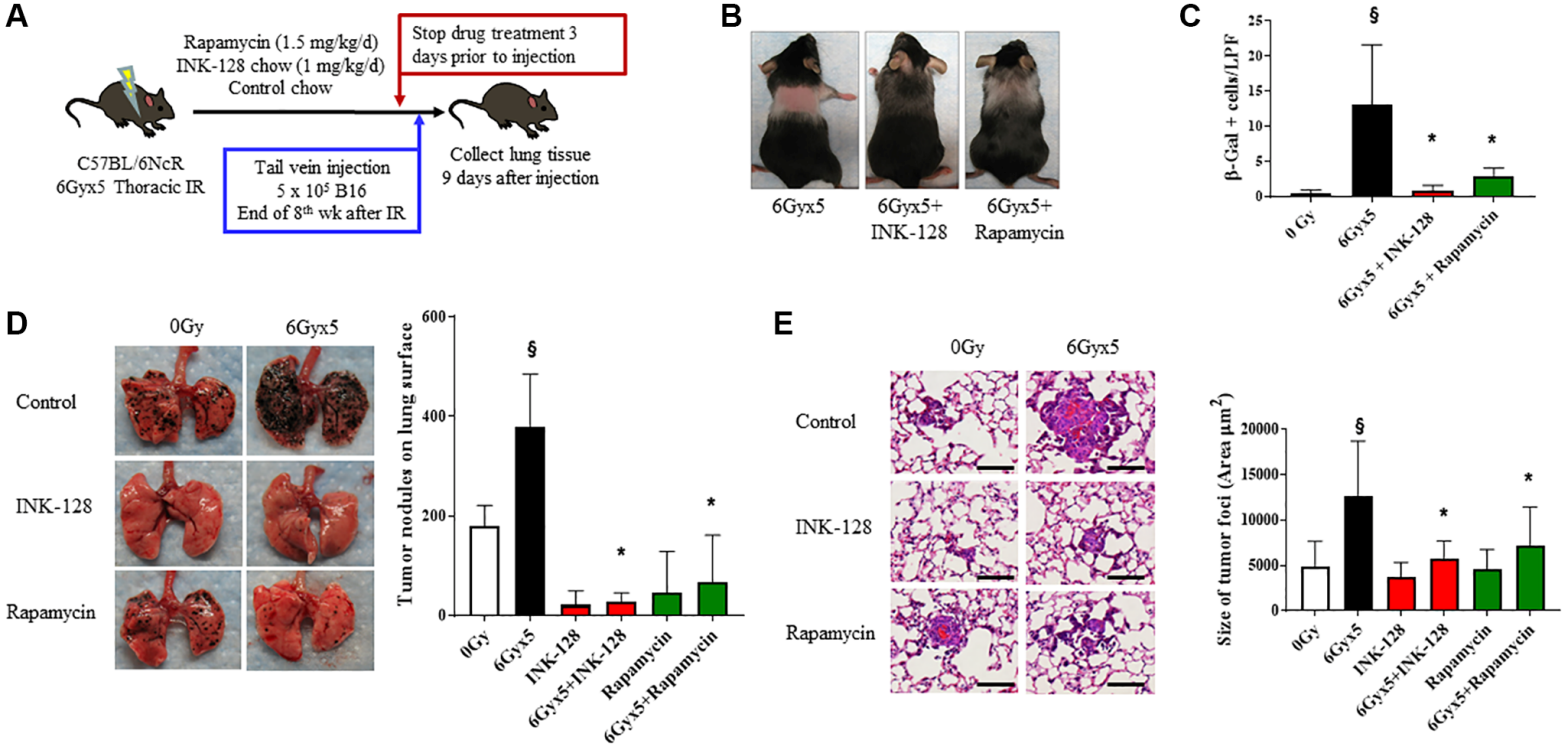
**Senostatic therapy with mTOR inhibition prevents IR-enhanced tumor growth.** (**A**) Experimental schema: Ten week old C57Bl/6NCr mice (*n* = 10 per condition) were exposed to no irradiation (0 Gy) or 6Gyx5 thoracic IR. Immediately after IR, mice were treated with Rapamycin (1.5 mg/kg/day), INK-128 (1 mg/kg/day), or control chow for 8 weeks. Three days after drug discontinuation, 5 × 10^5^ B16F0 cells were injected intravenously via the lateral tail vein. Lung tissue was collected 9 days later. (**B**) Coat color changes at 8 weeks after irradiation. (**C**) SA-β-Galactosidase positive cells were counted in lung tissue collected at 8 weeks post IR in mice that did not receive tumor injection. (**D**) Representative images and graph of lung tumor nodule counts. (**E**) Size of tumor foci in the lung, scale bar 80 μm, ^§^: indicates *p* < 0.05 compared to 0 Gy, Vehicle. ^*^: indicates *p* < 0.05 compared to 6Gyx5, Vehicle.

To assess whether preventing IR-induced senescence affected tumor growth, INK-128 or rapamycin treatment was initiated immediately after thoracic IR and continued for 8 weeks (Figure 3A). Mice treated with INK-128 or rapamycin demonstrated protection from IR-induced alopecia and graying seen in vehicle treated mice, an effect most pronounced with INK-128 (Figure 3B). There was no evidence of toxicity or weight loss in drug treated mice (Supplementary Figure 4A). Lung tissue from irradiated mice treated with INK- 128 or rapamycin had significantly fewer senescent cells compared to mice that were irradiated and treated with vehicle (Figure 3C).

Having shown that INK-128 and rapamycin prevented IR-induced senescence, we hypothesized that these agents would also prevent IR-induced tumor colonization and growth. Mice treated with thoracic IR were treated with INK-128 or rapamycin beginning immediately after IR and continuing for 8 weeks. Three days after drug discontinuation (>5 half-lives [34, 35]). B16F0 melanoma cells were injected in the lateral tail vein. Lung tissue was collected 9 days after tumor cell inoculation. INK-128 and rapamycin significantly reduced the number of tumor nodules in the lungs of irradiated mice compared to irradiated, vehicle-treated mice (Figure 3D). Tumor foci were significantly smaller in the INK-128 and rapamycin treated irradiated mouse lung when compared with vehicle-treated, irradiated controls (Figure 3E) suggesting significant protection from IR-induced tumor growth. Together, these data support the hypothesis that preventing senescence in irradiated lung reduces the enhanced tumor colonization and growth after IR.

### Senolytic therapy prevents IR-induced acceleration of tumor growth

**Figure 4:**
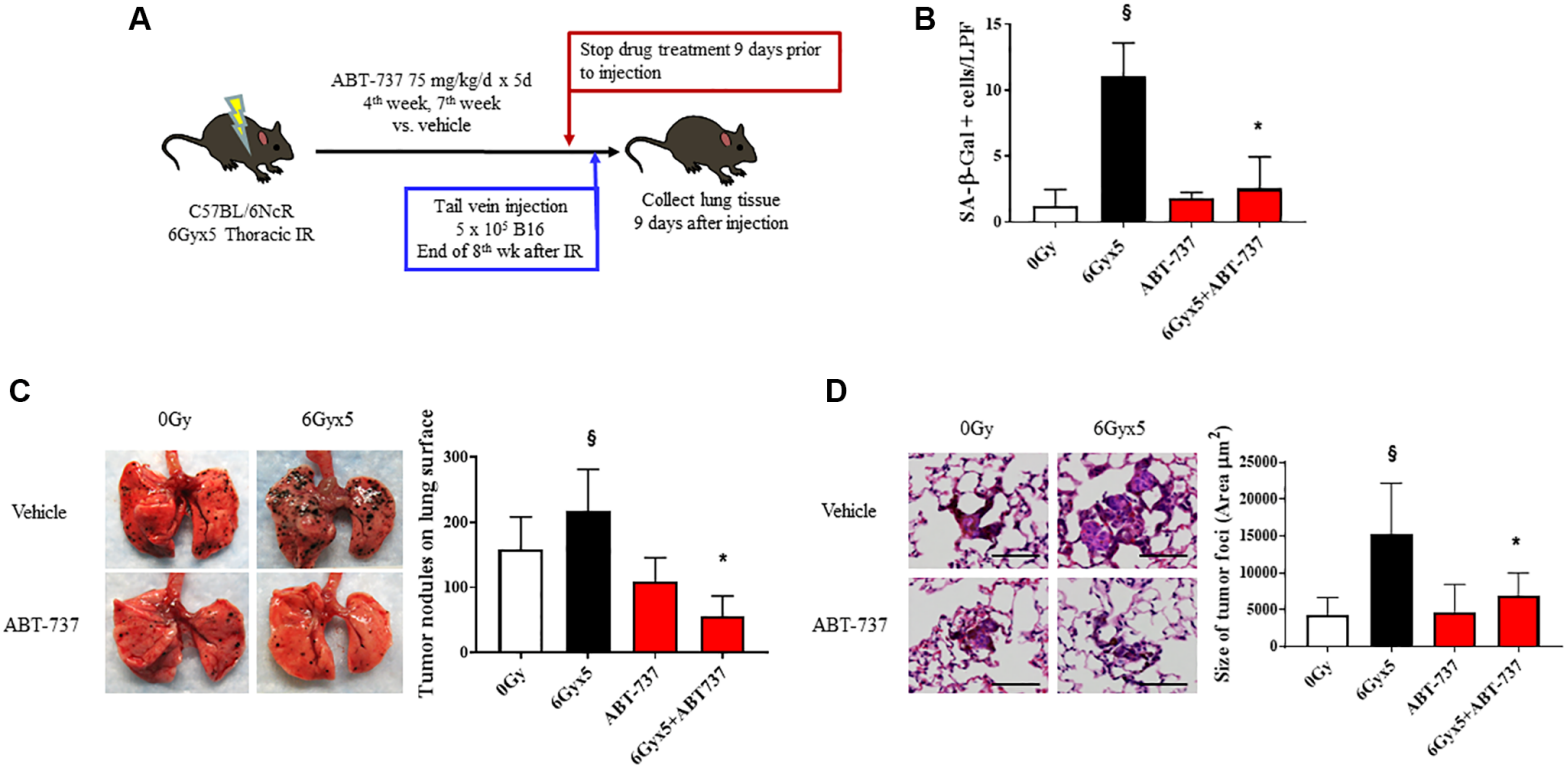
**Senolytic therapy with ABT-737 prevents IR-enhanced tumor growth.** (**A**) Experimental schema: Ten week old C57Bl/6NCr mice (*n* = 10 per condition) were exposed to no irradiation (0 Gy) or 6Gyx5 thoracic IR. Mice were treated with ABT-737 (75 mg/kg/day) or vehicle for 5 days during week 4 and week 7. Nine days after drug discontinuation, 5 × 10^5^ B16F0 cells were injected intravenously via the lateral tail vein. Lung tissue was collected 9 days after tumor inoculation. (**B**) SA-β-Galactosidase positive cells were counted in lung tissue collected at 8 weeks in mice that did not receive tumor injection. (**C**) Representative images and graph of lung tumor nodule counts. (**D**) Size of tumor foci in the lung, scale bar 80 μm, ^§^: indicates *p* < 0.05 compared to 0 Gy, Vehicle. ^*^: indicates *p* < 0.05 compared to 6Gyx5, Vehicle.

To confirm the importance of senescence in enhancing tumor growth after IR, we tested the impact of a senotherapeutic agent with a different mechanism of action in the same model. As noted in Figure 1 and prior publications [3], senescent cells accumulate in irradiated lung as early as 4 weeks after IR. To verify the senolytic activity of ABT-737 in irradiated lung, mice were treated with the senolytic agent ABT-737 in two 5-day cycles during the 4th week and 7th week after IR. There was no evidence of toxicity or weight loss in ABT-737 treated mice (Supplementary Figure 4B). Mice treated with ABT-737 demonstrated reduced pulmonary senescence and reduced radiation-induced alopecia and coat color changes compared to vehicle treated irradiated mice (Figure 4A, 4B). Based on these findings, the ABT-737 treatment regimen was combined with the experimental metastasis model in irradiated mice. ABT-737 treatment protected mice from IR-enhanced tumor colonization and growth emphasizing the important contribution of senescence in this phenomenon (Figure 4C, 4D).

### IR-induced acceleration of tumor growth requires 12-lipoxygenase

**Figure 5:**
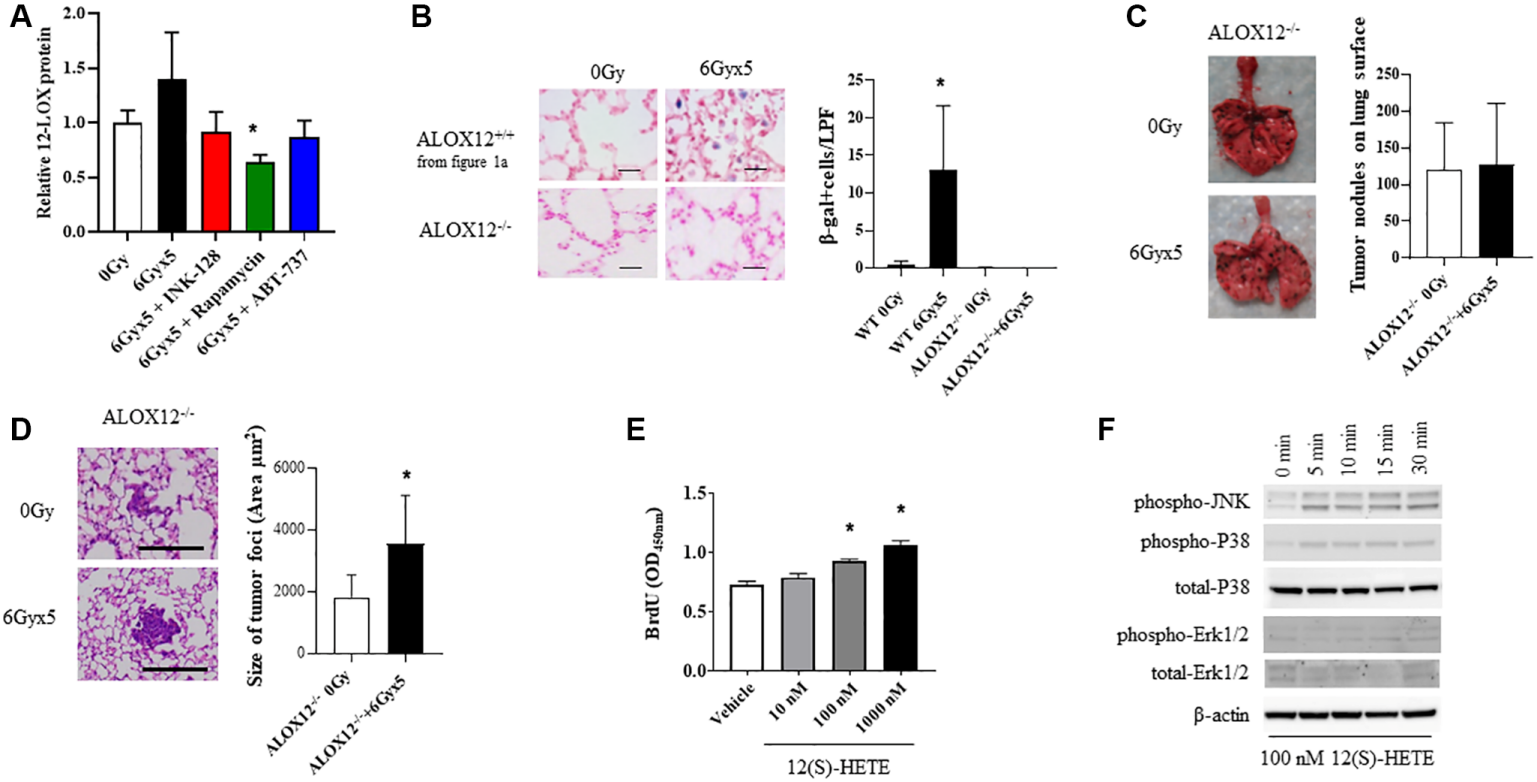
**ALOX12 deficient mice are protected from radiation-enhanced tumor colonization.** (**A**) Ten week old C57BL/6NCr mice (*n* = 3 per condition) were exposed to no irradiation (0 Gy) or 6Gyx5 thoracic IR alone or IR with Rapamycin, INK-128, or ABT-737. Lung tissue was collected at 8 weeks after irradiation (no tumor inoculation). The concentration of 12-LOX protein in lung homogenates was measured by ELISA. (**B**) SA-β-Galactosidase positive cells were counted in lung tissue collected from C57BL/6J ALOX12^−/−^ mice at 8 weeks after IR and compared to C57BL/6NCr ALOX12^+/+^ mice. (**C**, **D**) Ten week old C57Bl/6J ALOX12^−/−^ mice (*n* = 10 per condition) were exposed to no irradiation (0 Gy) or 6Gyx5 thoracic IR. Eight weeks after irradiation, 5 × 10^5^ B16F0 cells were injected intravenously via the lateral tail vein. Lung tissue was collected 9 days later to count tumor nodules on the lung surface (**C**) and to measure the size of tumor foci in the lung (**D**). (**E**) B16F10 cells were treated with 12(S)-HETE for a total of 48 hours. BrdU was added to cultures for the last 4 hours prior to assay of BrdU incorporation. (**F**) B16F0 cells were treated with 12(S)-HETE at the indicated doses and collected for Western blotting at 0–30 minutes.

Additional studies focused on 12-Lipoxygensae (12-LOX), a known contributor to radiation-induced senescence and lung injury were carried out [8]. 12-LOX metabolizes arachidonic acid to 12*S*-hydroperoxy-5*Z*, 8*Z*, 10*E*, 14*Z*-eicosatetraenoic acid (12(S)-HpETE), a SASP molecule that has been previously implicated in pulmonary senescence [8, 36, 37]. There was a trend towards increased expression of 12-LOX in lung tissue collected at 8 weeks after IR that was reduced when senotherapeutics were delivered with radiation (Figure 5A). After irradiation, 12-LOX is preferentially expressed in the relatively rare Type II airway epithelial cells relative to other pulmonary parenchymal cells (confirmed in Supplementary Figure 5) [8], likely diluting the concentration in whole lung. Conditioned media from senescent irradiated primary pneumocyte cultures was capable of increasing B16F0 proliferation and invasion relative to conditioned media from unirradiated primary pneumocyte cultures (Supplementary Figure 6).

To explore the role if 12-LOX and 12(S)-HpETE in radiation-enhanced tumor growth *in vivo*, additional studies were conducted in mice deficient in ALOX-12 (ALOX12^−/−^). In contrast to the robust senescent cell accumulation noted in wild type mice, SA β-Galactosidase positive cells were largely absent in irradiated lungs of ALOX12^−/−^ mice at 8 weeks after exposure (Figure 5B). Experimental metastases studies were then conducted in ALOX12^−/−^ mice, in which B16F0 cells were delivered intravenously 8 weeks after thoracic IR (Figure 5C). ALOX12^−/−^ deficiency significantly protected mice from radiation-enhanced tumor colonization, with the number of tumor nodules similar between irradiated and unirradiated ALOX12^−/−^ mice (Figure 5C). Tumor nodule size was increased with radiation (Figure 5D), but to a smaller degree than in wild type mice (less than two-fold).

To study this phenomenon further, B16F0, LLC, and A549 proliferation was measured in the presence of 12(S)-HETE, one of the end products of 12-LOX metabolism of arachidonic acid. 12(S)-HETE increased the proliferation of tumor cells in a concentration dependent manner (Figure 5E, Supplementary Figure 7). Treatment of B16F0 cells with 12(S)-HETE activated mitogenic signaling pathways, including JNK and p38 mitogen activated kinases (MAPK) (Figure 5F). Together these findings support a role for senescence associated 12-LOX activity in IR-induced tumor growth promotion.

## DISCUSSION

In this study, we demonstrate that thoracic irradiation can enhance tumor colonization and growth in lung, and that the magnitude of this effect correlates to the accumulation of senescent cells in lung after radiation exposure. Further, prevention of senescent cell accumulation or clearance of senescent cells in irradiated lung using agents with different mechanisms of action was sufficient to prevent radiation-enhanced tumor growth. Finally, we link senescence associated 12-LOX activity and production of 12(S)-HETE to the observed enhanced tumor growth after irradiation.

In this study, we utilized a lung metastasis model as a surrogate for tumor bed. The lung has a well characterized known chronology of senescence induction after radiation. An experimental metastasis model was also used to take advantage of the lung senescence chronology. Co-implantation of orthotopic models or tumor is not feasible in mouse models as the tumor grows rapidly after implantation, preventing adequate time for senescence to occur before lethality. In patients receiving radiotherapy, the tumor regrowth after radiation exposure often occurs much later, providing an opportunity for senescence to accumulate in the irradiated tumor bed. Thus, the experimental metastasis model was used to leverage the well-characterized timing of senescence in lung after radiation and allow accumulation of senescent cells before tumor inoculation. The SASP has been demonstrated to play an important role in tumor growth after chemotherapy [38]; however, the importance of the SASP on tumor growth after radiation and the importance of 12-LOX in this effect are less developed.

A major question in radiotherapeutic treatment is whether alterations in the tumor stroma and irradiated normal tissues that are adjacent to the tumor could stimulate or support tumor regrowth. The capacity of radiation to modulate tumor growth and metastasis has been attributed to alterations in the tumor bed vasculature, hypoxia, and pro-inflammatory cytokine release [6, 39, 40]. More recently, co-injection of previously irradiated fibroblasts has been demonstrated to enhance xenograft tumor growth due to elaboration of the SASP [19]. The induction of premature senescence in tumor stroma by radiation may contribute to these changes in tumor microenvironment and therefore the increase in recurrence and metastasis. Multiple molecules implicated in the radiated tumor bed effect or enhanced tumor growth after radiation, such as CXCR4, TGF-beta, IL-1 [4, 5, 19], are members of the SASP [37]. Collectively, these findings coupled with the efficacy of senotherapeutics in ameliorating radiation-enhanced tumor growth presented here support that senescence is a major contributor to the radiated tumor bed effect via the SASP.

mTOR signaling is essential for the induction of senescence, and dual mTOR complex inhibitors such as PP242 and Torin1 have been shown to suppress geroconversion, a transition of cell from a temporary cell cycle arrest to senescence [12]. In this context, we studied the previously unexplored senostatic efficacy of the dual mTOR complex inhibitor [41], INK-128, demonstrating for the first time that INK-128 effectively prevents radiation-induced senescence and radiation-enhanced tumor colonization and growth *in vivo*. In combination with the observed mitigation of radiation-induced alopecia and coat color changes, these *in vitro* and *in vivo* results provide compelling evidence for the senotherapeutic activity of INK-128. mTOR inhibition in tumor cells has been shown to impact metastatic colonization and tumor growth [41–43]. To exclude this as a possible mechanism for the observed effects in this study, drug treatment was halted more than 5 half-lives prior to tumor inoculation.

To provide additional supporting evidence of the role of senescence in radiation enhanced tumor growth, further studies were conducted with a senolytic agent, ABT-737, that possesses a different mechanism of action. Although rapamycin prevents senescence via inhibition of mTOR signaling, ABT-737 does not prevent senescence from occurring but instead selectively kills senescent cells through targeting of Bcl-2/bcl-xL, which is considered crucial for senescent cell survival [33]. Senolytic therapy has been shown to rejuvenate the hematopoietic stem cell pool (HSC) after total body irradiation [44] and to mitigate radiation lung injury through clearance of senescent cells [45]. The use of senolytics in animal models eliminates chemotherapy-induced senescent cells resulting in reduced therapy-induced bone marrow suppression, cardiac dysfunction, fatigue, and cancer relapse [38]. Herein, we confirm that the beneficial effects of senolytic therapy extend to prevention of radiation-enhanced tumor growth. Delivery of chemotherapeutics or other agents to mice can result in impaired oral intake. Caloric restriction may have a significant impact on tumor growth after treatment [46, 47], and is similarly implicated in tissue aging. To exclude caloric restriction due to drug toxicity as a confounder, animal health and animal weights were compared in irradiated mice treated with each agent evaluated here. There was no evidence of weight loss or evident toxicity with the drugs delivered.

12-LOX, the protein product of Alox12 transcription, and the downstream product 12-HETE, are expressed as a consequence of senescence and aging [8, 37, 48]. Thus, reduced expression of 12-LOX after treatment with senescence preventing drugs, such as rapamycin, or senolytics, such as ABT-737, is likely in part due to a reduction in the number of senescent cells. Further, several SASP molecules, such as Il-4 and IL-13, are known to increase the expression of 12-LOX [49, 50], possibly causing a positive feedback loop to further increase 12-LOX expression in the setting of senescence. Further, the release of arachidonic acid, the substrate for 12-LOX, has been shown in other models to increase after radiation exposure, thus possibly acting in concert with increased 12-LOX expression to increase 12-HETE production after radiation [51, 52].

12-HETE acts in an autocrine and paracrine manner, signaling through GPCR, GPR31 [53]. The 12-LOX-12HETE-GPR31 axis exerts pro-tumorigenic activity through activation of JNK, P38, and ERK [54, 55] which play a role in cancer cell proliferation and survival [56–58]. In this study, *in vitro* treatment of B16 cells with 12-HETE was confirmed to activate JNK and P38 pathways as well as increased cell proliferation. Prior studies have reported that co-injection of 12-HETE and B16 cells increases homing of melanoma cells to lungs, potentially due to increased adhesion of B16 cells to collagen, fibronectin and lung epithelium [59]. Recent work has implicated 12-LOX activity to other microenvironmental effects in irradiated tissue, such as altered macrophage recruitment and polarization and fibrosis [8]. Although fibrosis and macrophage polarization do not appear to play a major role in the effects observed in the studies presented here, it is highly likely that 12-LOX activity can impact the irradiated microenvironment in additional ways that remain unexplored.

The lung experimental metastasis model was utilized as a receptive tissue to study tumor growth relative to senescence, given the well-characterized progression of senescence after irradiation in lung [3]. The importance of the observations in therapeutic human exposures to irradiation remain uncertain, and these processes are most likely to impact local tumor growth after radiation. Further, the human therapeutic radiation exposures compared to mouse modeling differ in dose and fractionation, which result from species-based differences in radiation response. The required latency to observe senescence in mice is well-characterized but is uncertain in humans as obtaining irradiated non-tumor tissue for study is challenging. Recent work has demonstrated that senescence occurs in human lung after therapeutic radiation exposures, and that SASP molecules are expressed to a greater degree in irradiated lung [20]. To further address this concern, a human fibroblast line (WI-38) was used to confirm the senotherapeutic activity of rapamycin, INK-128, and ABT-737 in human cells. A human tumor line, A549, was noted to have similar response to 12-HETE exposure compared to B16 melanoma cells. Together, these findings support a potential clinical relevance of this study and support additional work to confirm these findings in humans and to optimize timing and drug delivery after radiation exposure to maximize effect.

Use of genetically modified mice deficient in 12-LOX activity allows an evaluation of the importance of this pathway without possible off target effects that are observed with molecular inhibitors. However, a small molecule inhibitor of 12-LOX activity would be a critical therapeutic tool for clinical translation. Additionally, the diverse repertoire of the radiation-induced SASP molecules and their complex interactions might govern multiple pathways that are important for radiation-enhanced tumor growth. Thus, targeting senescent cells in irradiated tissue may provide a multi-target approach to prevent radiation injury and target a tumor permissive microenvironment by suppressing multiple SASP molecules simultaneously. Towards this end, future work will explore the contribution of SASP components involved in regulating tumor growth in an irradiated lung environment.

Together, this study demonstrates the critical role of senescence in mediating radiation-enhanced tumor growth and identifies Alox12 as an important player in this phenomenon. Treatment with a senostatic agent, INK-128, identified in this study, or with agents like rapamycin and ABT-737 suggested their potential therapeutic use in alleviating radiation associated tumor growth.

## MATERIALS AND METHODS

### Cell culture

B16F0 (ATCC^®^, CRL-6322™) murine melanoma cells, murine Lewis lung carcinoma cells (LLC; ATCC^®^ CRL-1642™), NIH/3T3 (ATCC^®^ CRL-1658™) murine fibroblasts, A549 (ATCC^®^, CCL-185) human adenocarcinoma cells and WI-38 (ATCC^®^ CCL-75™) human fibroblasts were cultured in DMEM (Life Technologies, Carlsbad, CA, USA) containing 10% FBS (Corning^®^) at 37°C in a humidified incubator with 5% CO_2_.

### Animal studies

All mouse studies were institutionally approved and in accordance with the guidelines of the Institute of Laboratory Animal Resources, National Research Council. Ten-week-old female C57BL/6NCr mice (Frederick National Laboratory, Frederick, MD, USA), C57BL/6J mice (Jackson Laboratories, Bar Harbor, ME, USA) and B6.129S2-Alox12^tm1Fun^/J (*Alox12*^−/−^; Jackson Laboratories, Bar Harbor, ME, USA) mice were immobilized in a custom jig that allowed selective irradiation of the thorax and treated with no IR (0 Gy, control) or 6 Gy for 5 daily fractions (6Gyx5) using an X-RAD 320 X-ray irradiator (Precision X-Ray, Inc., North Branford, CT, USA) with 2.0 mm AI filtration (300 kV peak) at 2.61 Gy/min. Radiation dosimetry was confirmed by thermoluminescent dosimetry using Lucite phantom mice within the custom Lucite lung irradiation jig. For tissue-based studies, mice were euthanized at 8 weeks after IR and tissue was collected as described below.

For the experimental metastasis model, 5 × 10^5^ B16F0 or LLC cells were delivered intravenously via the lateral tail vein (*n* = 10 mice per group) at 2, 4, or 8 weeks after thoracic IR. Lung tissue was collected 9 days after B16F0 tumor injection, and 14 days after LLC injection, tumor nodules on the lung surface were counted and recorded. For LLC tumors, sagittal lung hematoxylin and eosin (H&E) stained sections were captured at 20 × magnification on an Olympus FSX100 microscope (Tokyo, Japan) and tumor nodules were counted. Portions of each lung were snap frozen in liquid nitrogen, frozen in OCT™ compound (Thermo Fisher Scientific™, Waltham, MA, USA), or inflated with neutral buffered formalin and paraffin embedded for histologic analysis.

In separate studies, mice (*n* = 10 per group) were irradiated (0 Gy or 5 daily fractions of 6 Gy thoracic IR) and treated with drug (INK-128, rapamycin, or ABT-737) or vehicle. INK-128 (DCTD, NIH, Bethesda, MD, USA) was delivered in chow (1 mg/kg BW/d) and rapamycin (LC laboratories, Woburn, MA, USA, 1.5 mg/kg BW/d) in drinking water beginning immediately after IR and continuing for 8 weeks. ABT-737 (Selleckchem, Houston, TX, USA, 75 mg/kg/d) or vehicle (10% DMSO, 30% propylene glycol, 5% Tween 80, 55% D5W) was delivered by intraperitoneal injection in 5-day cycles during the 4th and 7th week after IR. All drugs were discontinued for at least 5 half-lives prior to tumor injection (3 days INK-128 [34], 3 days rapamycin [35], 9 days ABT-737 [60]). Additional experimental details are included in the Supplementary Methods.

### Histology and tissue β-galactosidase activity

H&E stained lung sections were examined on a Leica DM LB2 microscope (Wetzlar, Germany) and tumor foci size was measured using Image J software. Masson’s Trichrome Stain (Sigma Aldrich) was performed per manufacturer’s instruction. Aniline blue staining was used to visualize collagen. To visualize senescence, frozen lung sections were evaluated with a senescence-associated β-Galactosidase activity assay (Abcam, Cambridge, MA, USA) according to the manufacturer’s instructions. Briefly, frozen tissue sections were thawed on ice, washed with cold PBS, fixed for 10 min and incubated with the staining solution overnight at 37°C. Sections were counterstained with Nuclear Fast Red (Vector Labs, Burlingame, CA, USA), dehydrated through graded alcohols to xylene, and mounted in Permount™ mounting media (Thermo Fisher Scientific).

### *In vitro* SA-β-galactosidase assay

Cells were plated in 4-well chamber slides and treated with INK-128, rapamycin, or vehicle beginning 6 hours after irradiation (0 Gy or 17.5 Gy). After 5 days, SA-β-Galactosidase activity was evaluated at noted above. In separate studies, ABT-737 or vehicle was delivered 3 days after IR and SA-β-Galactosidase activity was assessed after 2 additional days. The impact of drug and radiation treatment on SA-β-Galactosidase activity and cell numbers was assessed in at least 12 high power fields per condition/dose.

### 5-Bromo-2′-deoxyuridine (Brd-U) assay

Tumor cells were treated with 12(S)-HETE (Sigma-Aldrich, St. Louis, MO, USA) for 48 hours and proliferation was assessed with a BrdU assay kit (Millipore, Burlington, MA, USA) following the manufacturers’ instructions.

### Western blot analysis

B16F0 cells were starved of serum for 24 hours prior to treatment with vehicle (ethanol) or 12(S)-HETE. Cells were lysed in radioimmunoprecipitation assay buffer (RIPA, ThermoFisher Scientific) supplemented with Complete™ protease inhibitor cocktail and PhosSTOP™ phosphatase inhibitor cocktail (Roche). Protein concentrations were measured with BCA protein assay (ThermoFisher Scientific) and equal amounts of protein was separated by SDS-PAGE (NuPAGE, ThermoFisher Scientific). Proteins were transferred to nitrocellulose membrane (Trans-Blot, Bio-Rad) and subjected to immunoblotting with the following antibodies: Phospho-p44/42 MAPK (Thr202/Tyr204) or (Thr185/Tyr187), p44/42 MAPK, Phospho-SAPK/JNK (Thr183/Tyr185), Phospho-p38 MAPK (Thr180/Tyr182), p38 MAPK, (Cell Signaling Technology) β-actin (EMD-Millipore).

### 12S-LOX and 12-HETE ELISA

Lung tissue was homogenized in RIPA buffer containing protease and phosphatase inhibitors. Insoluble material was removed by centrifugation at 16,000g for 10 min. Protein concentration was measured using the BCA protein assay. 12S-LOX protein was quantified in samples using a 12S-LOX ELISA kit using the manufacturers protocol (MyBioSource, San Diego, CA, USA). 12-HETE was quantified in samples using a 12-HETE ELISA kit using the manufacturers protocol (Abcam).

### Statistical analysis

One-way analysis of variance (ANOVA) was performed with Tukey’s correction for multiple comparisons to compare differences between experimental groups, with a *p* value less than 0.05 considered statistically significant. *In vitro* studies were performed in triplicate and were repeated for validation. Tumor nodules were counted on the lung surface in all mice in each group (*n* = 10). Tumor size measurements were conducted in randomly selected fields (*n* = 10 mice, >12 measures per condition and time point). Cell counts were conducted in at least 5 randomly selected fields from each time point and condition (*n* = 5 mice).

## Supplementary Materials

Supplementary Methods

Supplementary Figures
