# Supplementary material for: Senescence-associated tumor growth is promoted by 12-Lipoxygenase
Source: Aging (Albany NY). 2022 Feb 14;14(3):1068–86. doi: 10.18632/aging.203890 (PMC8876904; doi:10.18632/aging.203890)
Supplement: Supplementary Methods [file aging-14-203890-s001.pdf]

SUPPLEMENTARY METHODS

Histopathology and histochemistry

For assessing collagen regulation, an indicator of fibrosis, paraffin embedded sections of the lungs were deparaffinized in xylene and rehydrated through graded series of alcohol to water. Sections were then incubated in Bouin's picric-formalin and then stained with aniline blue for collagen (Masson's trichrome Kit # HT-15, SIGMA). Sections were then dehydrated through graded alcohols to xylene, and coverslips were mounted with mounting medium (Permounte, Thermo Fisher Scientific). Slides were then observed on Leica DM LB2 microscope (Wetzlar, Germany), digital micrographs were captured and images were analysed on image J software for the analysis of staining intensity.

Immunohistochemistry was performed to identify specific macrophage populations in the experimental lungs. Briefly, paraffin-embedded lung sections were deparaffinized in xylene and rehydrated as mentioned before. Immunohistochemical assays were performed using antibodies to identify specific cell population, F4/80 (D2S9R XP® Rabbit mAb #70076S) for pan-macrophages and Arginase 1 (Arg1; ab118884, Abcam) for M2 macrophages etc. Compatible HRP tagged secondary antibodies were used and slides were developed with peroxidase substrate (DAB; Vector Labs). Slides were then dried, coverslips were mounted in permount and observed under the microscope. The number of each cell type was counted from five randomly selected fields from each lung.

Vascular permeability assay

FITC-Dextran (70 kDa) solution was intravenously injected in mice at 1 mg per animal. After exactly 30 minutes of injection, animals were euthanized, lungs were collected and perfused with PBS. Tissues were kept in dark, homogenized in RIPA buffer supplemented with protease and phosphatase inhibitors. Total protein content was then quantified using BCA protein assay (ThermoFisher Scientific). FITC-dextran in the lung homogenates was measured at 480 nm excitation and 530 nm emission wavelength. For quantifying the amount of FITC-dextran in each tissue, readings were interpolated on the FITC-dextran standard graph and expressed as ng per µg of protein.

Pneumocyte isolation and conditioned medium collection

Primary pneumocytes were isolated from 8 to 10-week-old C57BL/6 mice. Briefly, mice were anesthetized 10 minutes after intraperitoneal injection of Heparin Sodium (100 USP unit/mouse, Fresenius Kabi USA. LLC. Lake Zurich, IL, USA). Lungs were perfused with 10 ml of HBSS (containing 30 mM HEPES), filled with 1 ml enzyme cocktail (Elastase 3 u/ml, 0.01% DNase I and 0.2% Collagenase in HBSS containing 30 mM HEPES), and incubated in 5 ml of enzyme cocktail at 37°C for 30 minutes. The digested tissue was carefully teased from the airways and gently swirled for 5 to 10 minutes. The resulting suspension was successively filtered through 100 mm and 40 mm Falcon cell strainers, then centrifuged at 130 × *g* for 8 min at 4°C and resuspended in HBSS. The crude single cell suspension was applied to Ficoll density gradient isolation solution. Pneumocytes were collected from the layer of density 1.077~1.080, washed with HBSS, and then resuspended with DMEM media containing 10% FBS and 1% antibiotics. Enriched type II pneumocytes were plated and irradiated the following day with 0 Gy or 17.5Gy. Three days post IR, medium was replaced with the serum-free DMEM. Conditioned medium was collected after 24 hours and viable cell count was taken simultaneously. In parallel, pneumocytes were stained for pro-surfactant protein C (SPC-ab 3786) to confirm cell type. The volume of conditioned media was normalized to cell count for measuring 12-HETE.
