# Supplementary material for: Senescence-associated tumor growth is promoted by 12-Lipoxygenase
Source: Aging (Albany NY). 2022 Feb 14;14(3):1068–86. doi: 10.18632/aging.203890 (PMC8876904; doi:10.18632/aging.203890)
Supplement: Supplementary Figures [file aging-14-203890-s002.pdf]

SUPPLEMENTARY FIGURES

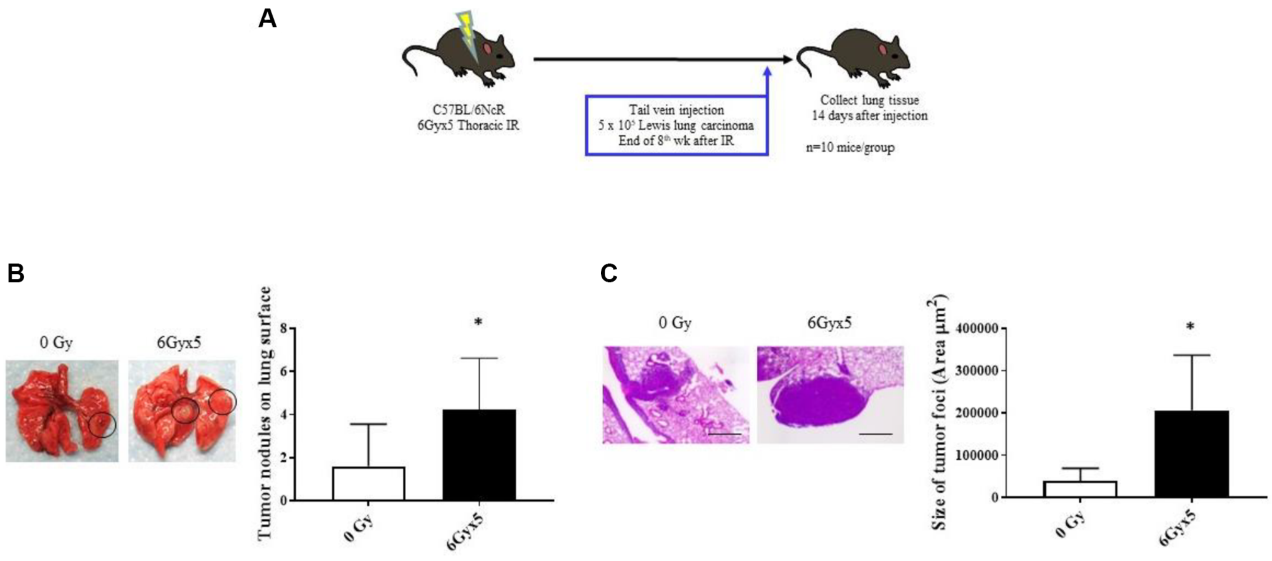

Supplementary Figure 1. IR enhanced tumor growth. (**A**) Experimental schema: Ten week old C57Bl/6NcR mice (n = 10 per condition) were exposed to no irradiation (0 Gy) or 6Gyx5 thoracic IR. Eight weeks after IR 5 × 10^5 LLC cells were injected intravenously via the lateral tail vein. Lung tissue was collected 14 days later. (**B**) H&E staining on sagittal sections of lungs from irradiated and/or drug treated animals that were injected with LLC tumor cells after 8 weeks post IR. *: indicates $p < 0.05$ compared to 0 Gy, Vehicle. (**C**) Tumor nodules on the lung surface. *: indicates $p < 0.05$ compared to 0 Gy, Vehicle

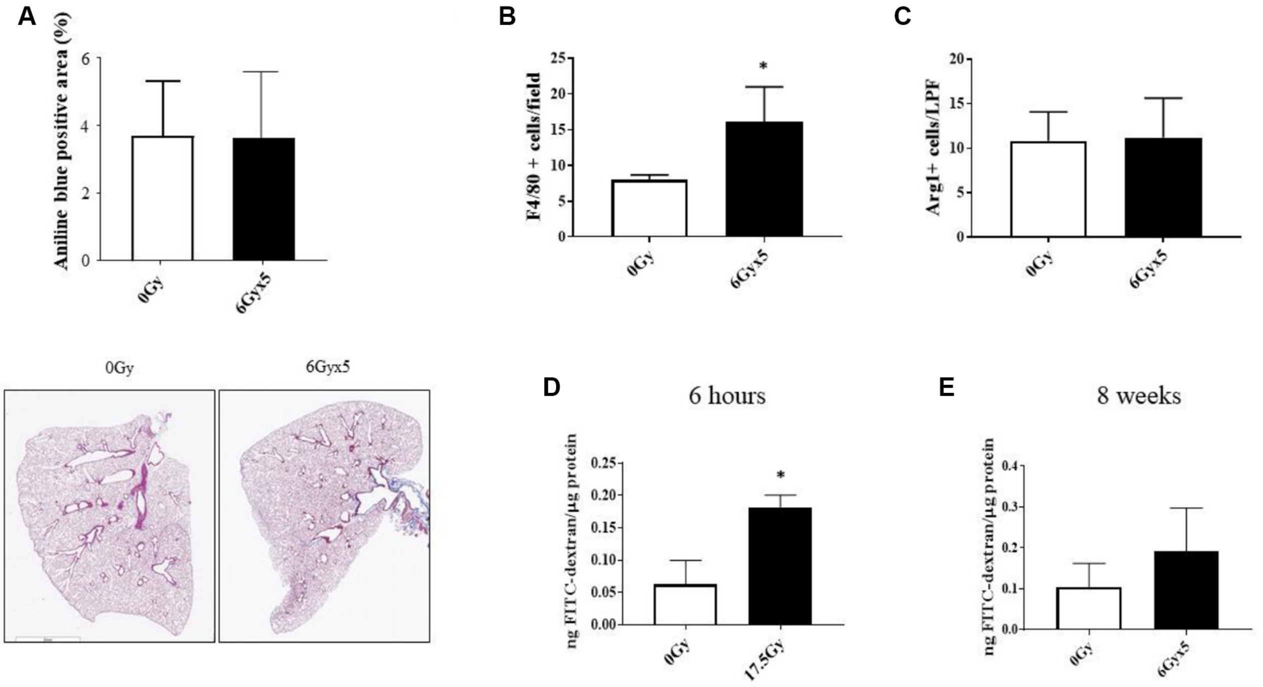

Supplementary Figure 2. Changes in lung tissue at 8 weeks after irradiation. Ten week old C57Bl/6NcR mice (n = 10 per condition) were exposed to no irradiation (0 Gy) or 6Gyx5 thoracic IR. (**A**) Lung tissue collected at 8 weeks after IR was stained with aniline blue and the intensity of collagen staining was quantified. Representative images of Masson's Trichrome staining are presented. No significant difference was observed in collagen content between the two groups. (n = 21 lung regions per condition). (**B**, **C**) Lung tissue collected at 8 weeks after IR was subjected to immunohistochemistry with (**B**) F4/80 specific antibodies (for pan-macrophages) or (**C**) Arginase 1 specific antibodies (for M2 macrophages) *: indicates $p < 0.05$ compared to 0 Gy. (n = 5 per condition). (**D**, **E**) Extravasation of 70 kDa FITC-dextran in the lung tissue was measured to estimate the changes in the vascular permeability in irradiated mice 6 hours after a single dose of 17.5 Gy thoracic IR (positive control) and 8 weeks after 6Gyx5 thoracic IR. *: indicates $p < 0.05$ compared to 0 Gy (n = 6 per condition).

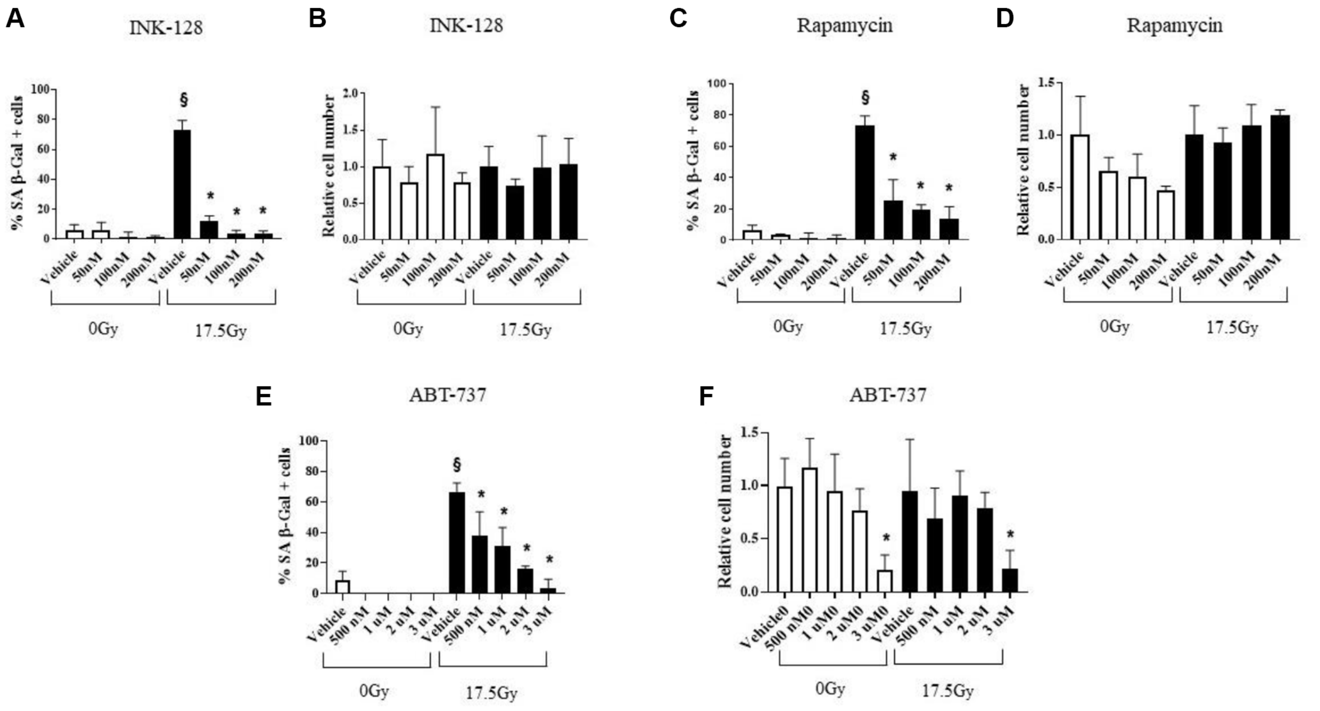

Supplementary Figure 3. Senostatic and senolytic action of drugs on human fibroblasts. WI38 cells were treated with a single dose of radiation (0 Gy or 17.5 Gy). (**A**–**D**) Six hours after irradiation, cells were treated with INK-128 or rapamycin. The percentage of cellular senescence, as determined by SA-β-gal activity, and relative cell number were assessed five days after drug treatment. (**E**, **F**) WI38 cells treated with a single dose of radiation (0 Gy or 17.5 Gy). Three days after irradiation, cells were treated with ABT-737. Two days after drug was applied the percentage of cellular senescence, as determined by SA-β-gal activity, and the relative cell number were assessed. Plating efficiency for each drug is normalized to vehicle from the respective group (0 Gy or 17.5 Gy). §: indicates $p < 0.05$ compared to 0 Gy, Vehicle. *: indicates $p < 0.05$ compared to 17.5 Gy, Vehicle.

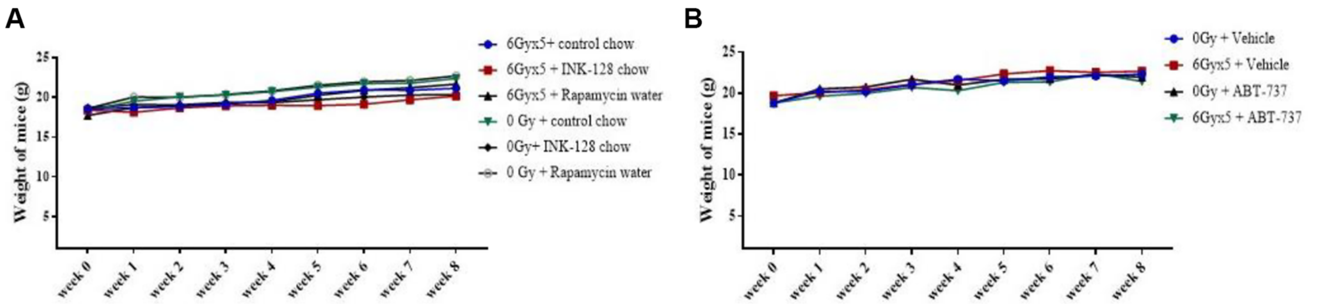

Supplementary Figure 4. Impact of IR, senostatic, and senolytic therapy on animal weights. Ten week old C57Bl/6NcR mice (n = 10 per condition) were exposed to no irradiation (0 Gy) or 6Gyx5 thoracic IR. Immediately after IR, mice were treated with (**A**) INK-128 (1 mg/kg/day), rapamycin (1.5 mg/kg BW/d), or control chow. Body weight was measured weekly, mean is presented. (**B**) Irradiated mice were treated with ABT-737 (75 mg/kg/day) or vehicle by intraperitoneal injection in 5-day cycles during the 4th and 7th week after IR. Body weight was measured weekly, mean is presented.

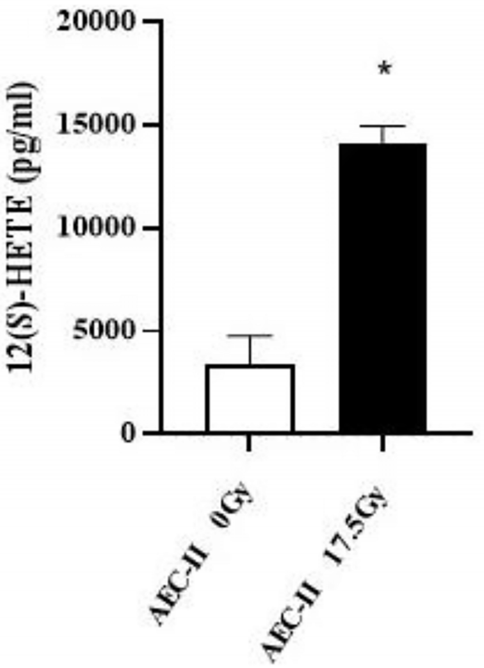

Supplementary Figure 5. 12-HETE production in AECII cells after irradiation. Primary type II pneumocytes (AECII) enriched from C57Bl/6NcR lungs were irradiated with 0 Gy or 17.5 Gy. Three days after IR, media was replaced with serum free DMEM. After 24 hours, 12-HETE production was measured in the media with ELISA. *: indicates $p < 0.05$ compared to 0 Gy.

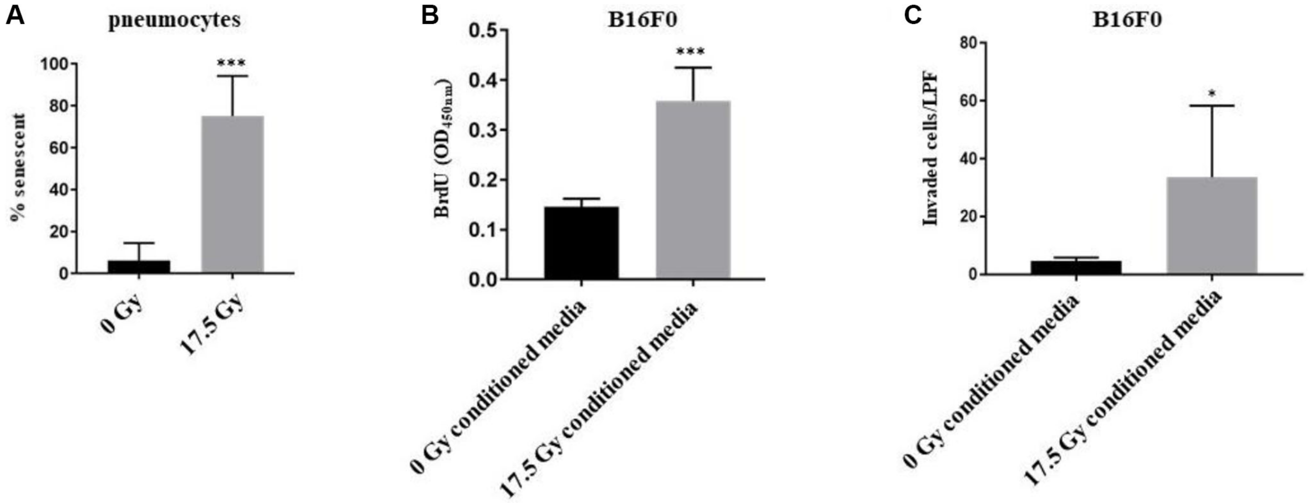

Supplementary Figure 6. Conditioned media from irradiated senescent primary pneumocytes stimulate B16F0 proliferation and invasion. Primary pneumocytes enriched from C57Bl/6NcR lungs were irradiated with 0 Gy or 17.5 Gy. Three days after IR, senescence was assessed (**A**). In parallel pneumocyte cultures, media was replaced with serum free DMEM 3 days after IR. After 24 hours, conditioned media was collected and applied to B16F0 melanoma cells to measure proliferation (**B**) or invasion in a Matrigel coated transwell chamber (**C**). * indicates $p < 0.05$ compared to 0 Gy, ***: indicates $p < 0.0005$ compared to 0 Gy. Abbreviation: LPF: low power field.

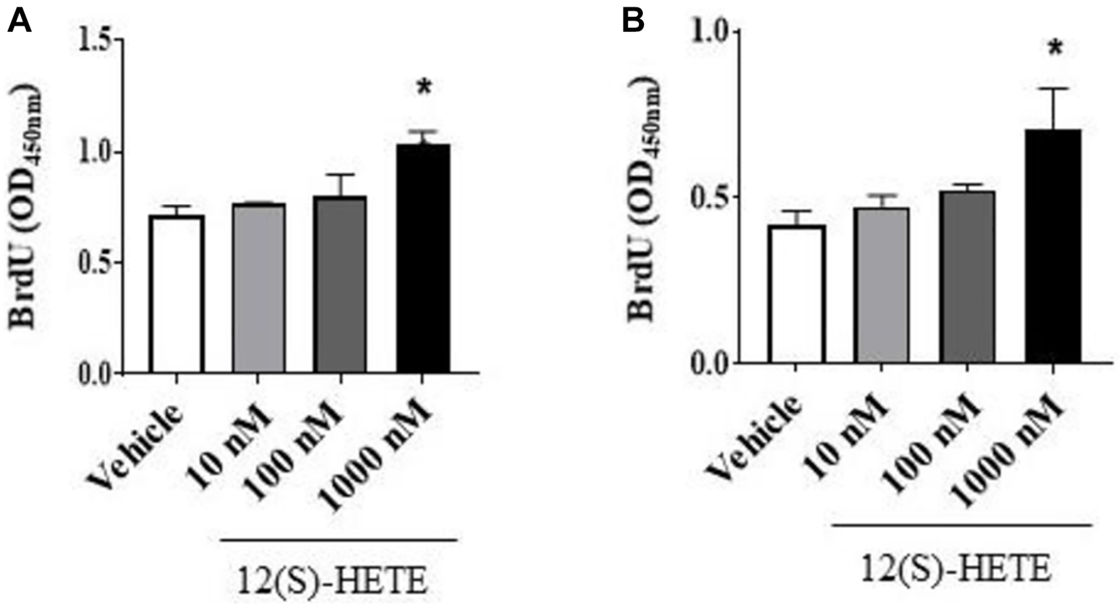

Supplementary Figure 7. 12(S)-HETE stimulates LLC and A549 cancer cell proliferation. LLC (**A**) and A549 (**B**) cells were plated in 96 well plates 24 hour before treatment with increasing concentrations of 12(S)-HETE. After 44 hours of 12(S)-HETE treatment BrdU was added to cultures. Differences in BrdU incorporation were assayed after 4 hours of incubation. *: indicates $p < 0.05$ compared to vehicle (ethanol).
